# Plasmonic Layer as a Localized Temperature Control Element for Surface Plasmonic Resonance-Based Sensors

**DOI:** 10.3390/s21062035

**Published:** 2021-03-13

**Authors:** Sivaramakrishnan Ganesan, Sophie Maricot, Jean-Francois Robillard, Etienne Okada, Mohamed-Taieb Bakouche, Laurent Hay, Jean-Pierre Vilcot

**Affiliations:** 1 Univ. Lille, CNRS, Centrale Lille, Univ. Polytechnique Hauts-de-France, Junia, UMR 8520—IEMN, F-59000 Lille, France; sivaramakrishnan.ganesan@univ-lille.fr (S.G.); sophie.maricot@univ-lille.fr (S.M.); jean-francois.robillard@yncrea.fr (J.-F.R.); etienne.okada@univ-lille.fr (E.O.); mohamed-taieb.bakouche@univ-lille.fr (M.-T.B.); 2 Univ. Lille, CNRS, UMR8523-PhLAM-Physique des Lasers Atomes et Molécules, CERLA/IRCICA, F-59000 Lille, France; laurent.hay@univ-lille.fr

**Keywords:** surface plasmon resonance, plasmonic sensor, temperature control, localized heating

## Abstract

Surface plasmon resonance (SPR) sensing is a well-established high-sensitivity, label-free and real-time detection technique for biomolecular interaction study. Its primary working principle consists of the measurement of the optical refractive index of the medium that is in close vicinity of the sensor surface. Bio-functionalization techniques allow biomolecular events to be located in such a way. Since optical refractive indices of any medium varies with the temperature, the place where the measurement takes place shall be within a temperature-controlled environment in order to ensure any temperature fluctuation is interpreted as a biomolecular event. Since the SPR measurement probes the sensed medium within the penetration depth of the plasmonic wave, which is less or in the order of 1 µm, we propose to use the metallic film constituting the detection surface as a localized heater aiming at controlling finely and quickly the temperature of the sensed medium. The Joule heating principle is then used and the modeling of the heater is reported as well as its validation by thermal IR imaging. Using water as a demonstration medium, SPR measurement results at different temperatures are successfully compared to the theoretical optical refractive index of water versus temperature.

## 1. Introduction

Since the first demonstration of the surface plasmon resonance (SPR) phenomenon as a tool for probing the electrochemical behavior at interfaces, more than 40 years ago [1], the technique has been extensively studied taking advantage of the various developments in its different constitutive technological areas: plasmonics, optics, fluidics, surface functionalization, and data processing. It has also been applied to a great variety of application domains in chemical or biological sensing [2]: environmental monitoring [3], clinical diagnostics [4], food safety screening [5], drug discovery [6], and, of course, measurement of optical constants [7,8] since it is its basic functioning principle.

The physical principle of SPR-based measurements indeed relies upon the excitation of the so-called surface plasmonic wave at the interface between two different materials. Plainly explained by Fano in 1941 [9], who reexamined the first observations of light diffraction anomalies obtained on metallic gratings by Wood as of 1902 [10], the sustaining theory for the excitation of surface plasmon polaritons or surface plasmonic waves has since been widely and extensively described e.g., [11]. We do not intend to rephrase this theory here; we will just remind the reader of several basic conditions that have to be fulfilled in order surface plasmonic waves to be created. First of all, the real part of the permittivity of both materials, i.e., the sensed medium and the sensor surface, needs to be of opposite signs. Since sensed media, such as biological, chemical, or even gas solutions, have a positive sign, the sensor surface needs to be made of a metal presenting a negative real part at the excitation wavelength. As the latter is usually within the visible or the near-IR range, gold and silver are usually cited as the major, but not the only, players for that use. Gold is, however, usually used since silver is quickly oxidized when exposed to the sensed media. Secondly, the coupling condition ensuring the matching of the propagation constant of the plasmonic and excitation light waves is commonly fulfilled by means of an attenuated total reflection method, the so-called Kretchmann’s configuration [12], which uses a prism. Another prism coupling technique exists, the Otto’s configuration [13], but it is not often employed since it is of much less practical implementation for biosensing. As originally observed by Wood, coupling can also be achieved using a grating, but its fabrication cost is much higher than that of a prism, which is why this method is not often applied. Thirdly, the resolution of Maxwell’s equations for this metal-dielectric interface (corresponding to the sensor surface-sensed medium) does not provide any solution for s-polarized (TE) modes, and only p-polarized (TM) modes can generate the plasmonic wave that is evanescent in the direction either along the propagation (parallel to the interface) or perpendicular to it (perpendicular to the interface, so within the sensed medium and also the metal); those give rise, respectively, to the notions of propagation length and penetration depth [11].

**Figure 1:**
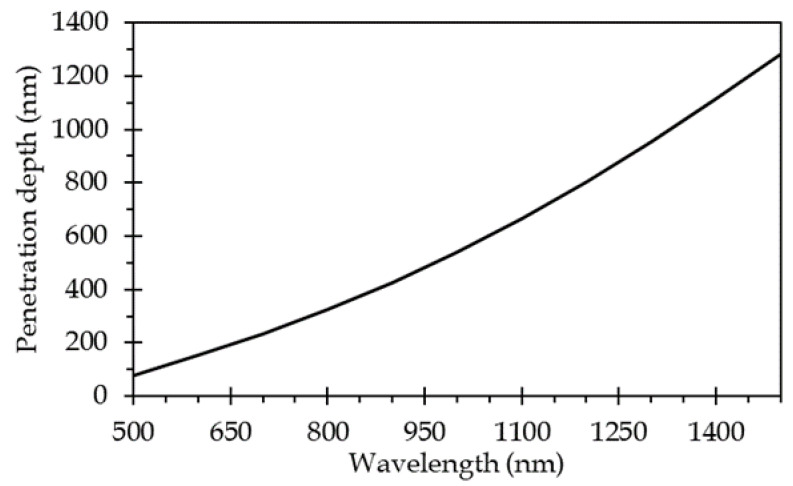
Penetration depth of plasmonic wave into the sensed medium (here water) versus wavelength of excitation light beam (here, the sensor surface is composed of 35 nm of gold).

This last characteristic is governing the use of the SPR measurement technique as to determine the permittivity, and thus the refractive optical index of the medium, which is in very close proximity of the sensor surface, more precisely within the penetration depth of the plasmonic wave into this medium. This penetration depth has been calculated over the 500–1500 nm wavelength range (Figure 1). It can be noticed that it varies by roughly one decade, for an excitation wavelength between 500 nm up to 1500 nm, over this wavelength range. Thus, it can be noted that the SPR measurement is blind to any event arising outside the penetration depth, i.e., roughly 1 µm above the sensor surface if the full wavelength range is considered.

In order to ensure molecular interactions can be detected, they need to occur within this distance from the sensor surface. Surface functionalization techniques are then used to convert the original sensor surface into a biomolecular interacting surface for the desired application [14]. The sensor is, thus, converted into a biosensor that will convert any biomolecular event arising at sensor surface into an optical refractive index variation. However, whatever the medium at sensor surface is, its optical refractive index will depend on the temperature, which, thus, modifies the measurement [15]; SPR has then understandably been investigated as an optical temperature sensing technique [16,17]. Consequently, any of the smallest temperature alteration can be interpreted as a molecular interaction, and thus, give a false biosensing result. 

Moreover, temperature has already been determined as a sensitive parameter for either DNA–DNA or antibody–antigen interactions. Denaturation of DNA is a well-known example of the influence of temperature [18]. Then, double-strand DNA molecules can be separated into two single strands by heating. This so-called melting temperature depends on the DNA strand composition and length and is within the range of 60 to 90 °C [19]. Flexibility of DNA molecules in the 25–65 °C temperature range has been investigated [20] and temperature-activated DNA structure modification has also been studied on the binding affinity to proteins from ambient temperature to 90 °C [21]. The temperature dependence of DNA–DNA and DNA–RNA hybridization and melting has also been studied by the surface plasmon resonance technique using a heated metallic support [22]. Concerning most cases of antibody-antigen interactions, association and dissociation rates have been shown to follow basic thermodynamic principles, and temperature then governs the biomolecular interaction constants [23]. Using the isothermal titration calorimetry technique, the protein binding affinity to hydrophobic adsorbent has been shown to be largely increased with temperature in the 20–60 °C temperature range [24]. Using the same technique, this binding affinity between monoclonal antibodies and antigens has been recorded at higher value for 40 °C compared to 37 or 42 °C [25]. Using a specifically designed temperature-jump electrospray-ionization source for the mass spectrometry measurement technique, it has been shown that precise temperature control as well as temperature jumps on a temperature range between 20–75 °C can give valuable information on DNA and protein molecular interaction behaviors [26]. Finally, a temperature-controlled measurement chamber has been used to conduct the LSPR measurements on the deformation of adsorbed vesicles on solid supports [27]. Those few examples show that the temperature greatly influences the molecular interactions and that their study requires the temperature to be controlled and monitored; for commercial SPR systems, this is usually done using a temperature-regulated enclosure, as used for the LSPR experiment in [27].

Drawing on the fact that only the space contained within the penetration depth of the plasmonic wave is sensed, we proposed a new way for managing the temperature during molecular interaction experiments by using the plasmonic layer as a Joule heater, which will provide a localized temperature source. The Joule heating technique has several interesting features, such as a rather simple implementation, real-time temperature modulation, heat localization, low cost, and miniaturization. As can be noticed in the above-mentioned examples of biomolecular event studies, those are commonly conducted in the 20–90 °C temperature range, 90 °C being the highest value that has been recorded. Most studies are located within the 37–42 °C range, which corresponds to human body temperatures and some require up to 60–70 °C for particular DNA–RNA interactions. Obviously, the Joule heating technique can only be used to adjust the temperature above the ambient one, which is, in the majority of cases, what is being sought. 

Following the common features of SPR-based measurements mentioned above, when using the Kretschmann’s configuration, SPR sensors are usually made of a plain metallic surface deposited on a glass support that is of the same material, i.e., same refractive index, as the coupling prism in order avoiding any parasitic reflections. In order to demonstrate the heat localization asset, the plasmonic layer shall be spatially delimited for controlling the current path. We decided to delineate four electrical wires that have the same fingerprint than the fluidic channels that are used.

The heating behavior has been simulated under COMSOL Multiphysics ® and experimentally validated by thermal IR imaging. The first demonstration of SPR measurements on a water drop that is heated in such a manner is reported. The refractive index of the water drop is correctly measured up to temperature rises of some tens of degrees. Such temperature rises are enough to check the action of the temperature on molecular interactions.

## 2. Design and Fabrication of a Multichannel SPR Sensor

### 2.1. Design and Fabrication

The SPR sensor is fabricated on a glass slide (H-ZF1 in our case) that is compatible with the SPR test set-up that is employed (see Section 5), which uses such a glass type coupling prism. Obviously, the use of any other glass substrate is possible; it does not affect the principle that is presented herein. The plasmonic layer shall be patterned to delimit the current path and so control the heating process. Thus, each sensing channel is materialized by a separate plasmonic layer. For this demonstration purpose, a basic four-channel design is applied. A lithography process is used to delineate the four channels. The glass substrate is initially cleaned using a piranha solution (H_2_SO_4_: H_2_O_2_). This is followed by the deposition of AZ1512 (1.5 µm height) photo-resist film. A laser lithography system (DILASE 650 from Kloé) is used to insulate the photo-resist film. After the development of exposed parts, a lift-off process is made to implement the plasmonic layer constituted of a Ti/Au film (2 nm/35 nm; Ti is used as an adhesion layer) that is deposited by e-beam evaporation in a high vacuum system. The deposition rate is 0.2 nm/s. 

**Figure 2:**
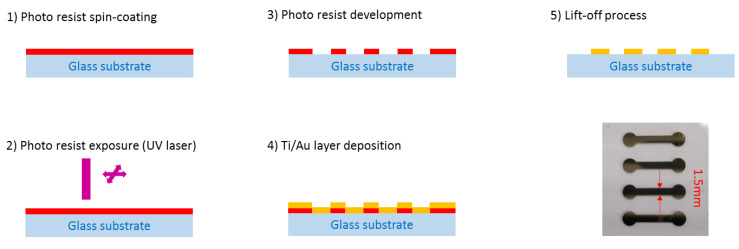
Process flow for the fabrication of the plasmonic sensor. View of the four-channel surface plasmon resonance (SPR) sensors (bottom right).

Figure 2 summarizes the main steps of the fabrication process. A top view of the fabricated sensor is shown on bottom right of Figure 2. Each channel is 1.5 mm wide and 1 cm long. Channel separation is 4 mm, from edge to edge.

### 2.2. Characterization of the Electrical Properties of the Plasmonic Layer

**Figure 3:**
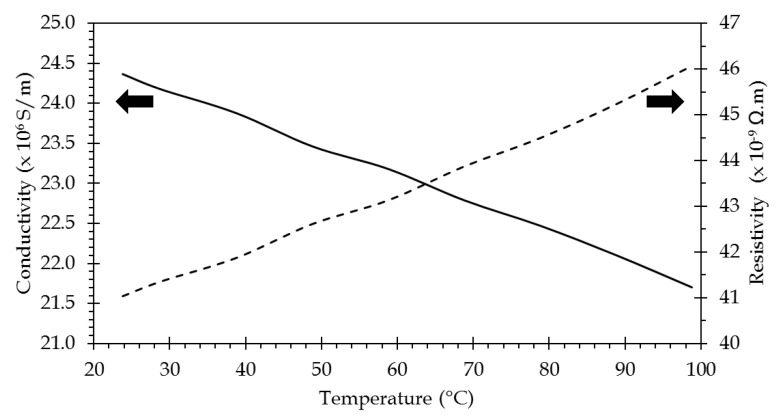
Electrical properties of the plasmonic layer gold film versus temperature.

Since the resistivity of the plasmonic layer will directly impact the resistance of the channel, it has been measured versus the temperature. The sensor has been placed on the hotplate of a thermal measurement system (see Section 4.1) and the resistance of a channel has been measured in a classical four probe scheme. Taking into account the dimensions of this channel (see Section 2.1 above), the resistivity (and conductivity) of the plasmonic layer has been calculated. The result is shown in Figure 3. In the 20–100 °C, resistivity value is between 41 and 46 nΩ.m, which is in very good agreement with values reported for such thin films [28,29]. The value of the temperature coefficient of resistivity (TCR) has been determined as 1.65 × 10^−3^/°C, which is in good agreement with the reported values of TCR for evaporated thin films of gold on glass [30]. This value of TCR is used to extrapolate the resistivity value to higher temperatures.

## 3. Modeling of the Sensor Behavior

### 3.1. Temperature

#### 3.1.1. COMSOL Modeling

**Figure 4:**
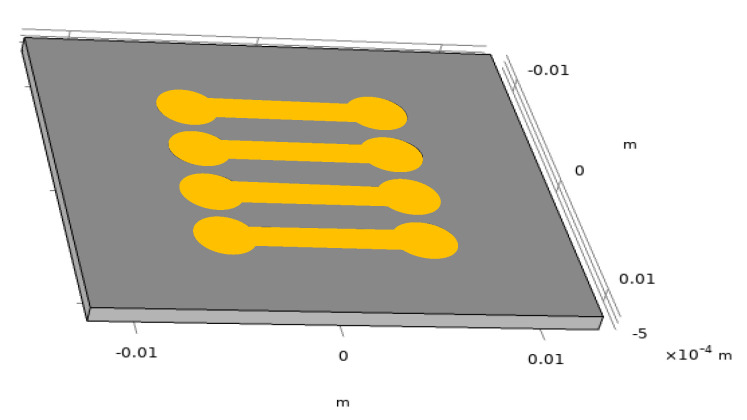
COMSOL simulated structure (metallic SPR surfaces are symbolized by yellow color).

COMSOL has been used to simulate the thermal behavior of a channel under current injection. The simulated structure is presented in Figure 4. In a first attempt, the full sensor has been simulated. The glass substrate is 2.5 cm × 2.5 cm and 1 mm thick and the four channels have been implemented. The simulation parameters for both the glass substrate and gold are shown in Table 1. 

The first three parameters are used for simulating the thermal behavior and the last one for the electrical one. Owing to the determination of the metallic film conductivity versus temperature (see Section 2.2), the resistance of the film is varied according to temperature. The atmosphere has been set to air and ambient temperature to 20 °C. A heat transfer coefficient value of 10 W/(m^2^.K) has been used. It corresponds to the mean value of this parameter, which is usually taken between 5 and 25 W/(m^2^.K) for natural convection in an infinitely large volume of air [31].

**Figure 5:**
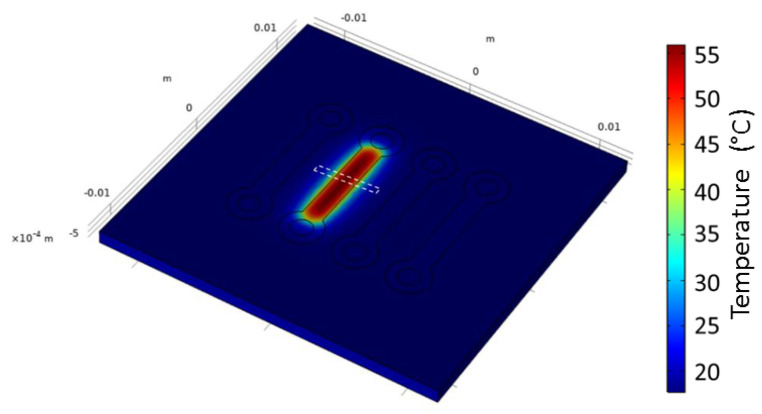
Typical 2D heatmap of sensor surface temperature under current injection (here, 300 mA). The white delimitation refers to the narrowed surface area that will be used in further modeling (see Figure 6).

**Figure 6:**
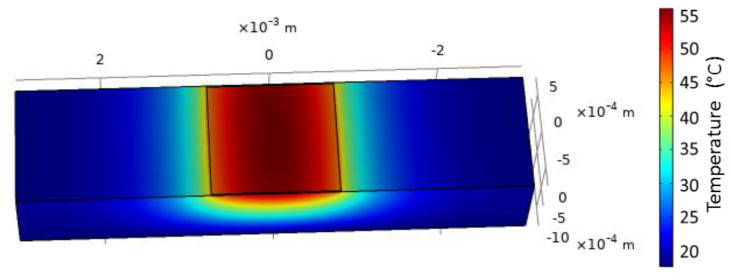
Narrowed surface area modeling window, under the same current injection conditions as the full sensor (see Figure 5).

The current has been injected into one channel. A 2D heatmap is shown in Figure 5. As will be detailed afterwards (see Figure 6), lateral thermal expansion is limited to the close vicinity of the channel. This has two main repercussions:

- It consolidates our original goal having independent control of each channel;
- It allows us to narrow the modeling window (white delimitation in Figure 5), and thus, considerably decrease the calculation time.

Thus, the modeling was replicated in a smaller window (Figure 6): the extension from the channel edge is 2.25 mm on each side of the channel width and its length is 1.5 mm (full window is 6 mm wide and 1.5 mm long). The current injection is made up of the full width of the channel (1.5 mm). The temperature that is obtained in such a modeling configuration is the same as obtained on the full sensor, and this result validated such calculation window narrowing for further modeling. 

**Figure 7:**
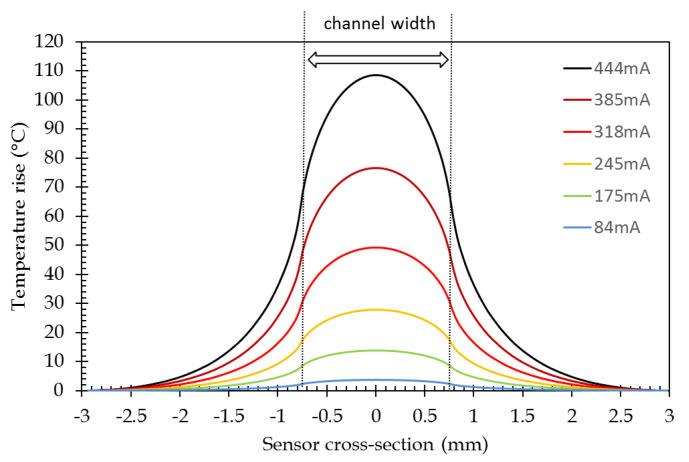
Rise of temperature-induced by the current injection (sensor cross-section: 0 corresponds to the channel center).

The lateral temperature profile across the channel section at sensor surface is reported in Figure 7. High-temperature rises can be obtained, but for practical cases, e.g., biological interactions, temperature rises, mainly in the order of some tens of degrees, will be of concern (see Section 1).

A temperature gradient can be observed along the channel width exhibiting a higher temperature at the channel center than on the channel edges. Such a temperature gradient is quite classical for flat wires and has already been described for PCB lines [32] or integrated circuit interconnections [33]. Here, we observed that this particular plasmonic layer is very thin and deposited on glass. This temperature inhomogeneity over the channel width increases with the temperature and the bell-shaped aspect of the curve is more and more pronounced. 

**Figure 8:**
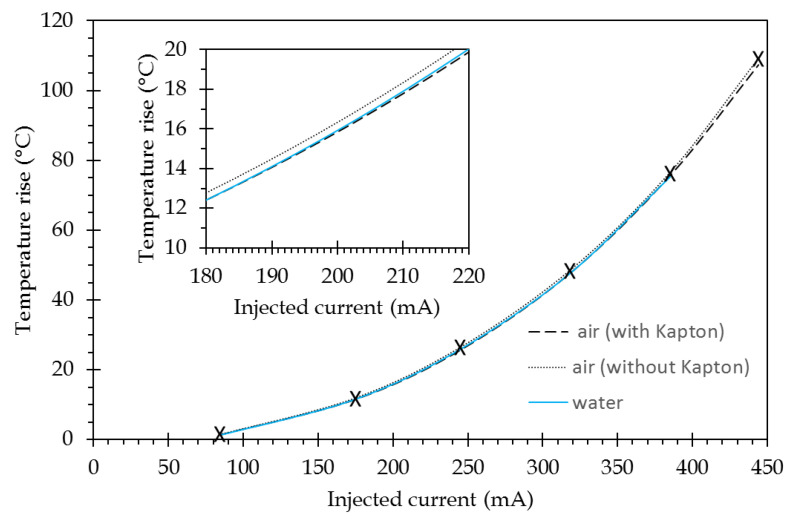
Comparison of the temperature rise versus the injected current for different experimental conditions: air without Kapton and air with Kapton for temperature measurements, and water for SPR measurements. Crosses indicate modeling points, while lines are the polynomial approximations that are used in the modeling (see Section 3.2.2). The insert is a zoom exhibiting the slight differences between those different cases.

The maximum temperature rise (value at the channel center) versus the injected current is reported in Figure 8 (without the Kapton curve). 

#### 3.1.2. Inclusion of Experimental Conditions into the Modeling

##### Thermal Camera Measurements

As will be discussed in the experimental section dedicated to thermal measurements, a 50 µm-thick polyimide film (Kapton) has to be inserted for temperature measurements by infrared flux detection. To check the impact of such a film on the temperature that is measured, it has been inserted in the modeling. A block representing this film is stacked over the full surface area. Its parameters are: ρ = 1300 kg/m^3^, κ = 0.25 W/(m.K) and Cp = 1000 J/(Kg.K) [34]. The modeling has then been conducted in the same way as previously. Figure 8 shows the results for both uncovered (without Kapton) and covered (with Kapton) channels; for both cases, the temperature is recorded at the top surface, i.e., on the metallic layer for the uncovered case and on Kapton film above the metallic layer for the covered case. For the latter, the temperature is slightly lower than for the uncovered case. A mean variation value of −1.8% is obtained over the full temperature range. During the measurements that needed to be made using a Kapton film, the real temperature value at sensor surface can be deduced by affecting the measured values with this coefficient.

##### SPR Measurements

For the SPR measurements, a water drop mimicking a biological solution has been set on sensor surface. It has been represented as a circular disk with a 1-mm radius and 50-µm thickness on top of the metallic surface in the COMSOL modeling. The water parameters are: ρ = 998.2 kg/m^3^, κ = 0.58 W/(m.K), and Cp = 4183 J/(Kg.K) @ 20 °C [35]. The same modeling as above was reiterated; its results are presented in Figure 8. For water, the current values have been limited, since higher current values give rise to unrealistic experimental temperature conditions leading to evaporation and boiling. For the modeled thickness of the water drop (50 µm), the behavior is rather similar to the one obtained for the air environment with a Kapton film.

**Figure 9:**
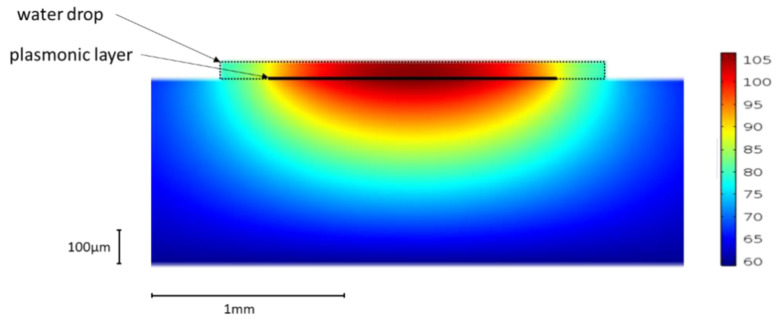
2D heatmap of the sensor. The injected current is 318 mA. The dotted line symbolizes the water drop contour and the thick line the plasmonic layer.

The 2D heatmap (Figure 9) shows the temperature gradient around a heated line. Although it is hardly perceptible, a very slow temperature decrease is observed in the direction perpendicular to the sensor surface all over the simulated thickness (50 µm). Moreover, it has to be recalled that the penetration depth of the plasmonic wave within the sensed medium is considerably lower since it is around 300 nm at 800 nm wavelengths (see Figure 1). Within this distance, let us say 1 µm, from the metallic heating film, no variation in the temperature can noticeably be pointed out. It can then be considered that the temperature of the sensed medium in a direction perpendicular to the surface is identical to that at the metallic film surface.

### 3.2. SPR Model

A common transfer matrix algorithm was used for the modeling of the SPR phenomenon [36]. The model can handle up to 10 layers. Nevertheless, two less common issues were inserted in the model to adapt to the proposed application, i.e., the effects of current injection and temperature. 

#### 3.2.1. Current Injection

**Figure 10:**
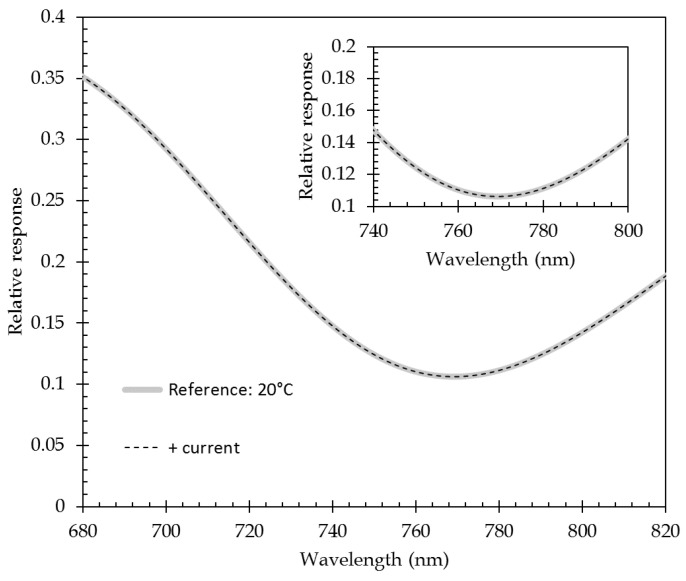
Effect of current injection (here, 318 mA) on the SPR response curve. In insert: zoom of the minimum of the response curve.

In [37], the use of current injection into a plasmonic layer was basically evaluated, imparting a unidirectional propagation direction to surface plasmonic waves. This modeling approach is based on the theory of fluctuations in plasmas [38] and proposes taking into account the effect of injection current via the drift velocity it induces on electrons. This drift velocity then acts as a Doppler frequency shift on the frequency *ω*, which is then considered as *ω’*:

$$\omega^{\prime }\equiv \omega -k.v$$

where *k* is the wavevector and *v* the drift velocity engendered by the injection current, *I*, as:

$$v=\frac{I}{q*n*S}$$

where *q* is the electronic charge, *n* the free-electron density in gold (≈5.9 × 10^22^ cm^−3^), and *S* the section of the metallic channel. Applying this approach to the plasmonic layer that is used here, of which the section is 52.5 × 10^−12^ m^2^ (35 nm × 1.5 mm), the current density is 5.7 × 10^9^ A/m^2^ for an injected current of 300 mA. The corresponding drift velocity and Doppler frequency shift are then close 0.6 m/s and 2.5 × 10^7^ s^−1^. This latter is, at least, 10^−8^ times lower than the value of signal angular frequency (around 2.5 × 10^15^ s^−1^). This frequency shift is then introduced in the Drude model of the metal permittivity. The Drude model that is used here (either for Ti or Au) uses the parameters given in [39]. Figure 10 shows the modeling result that has been obtained for a current injection of 318 mA: no perceptible modification of the response curve is observed. For this model, only the modification of metal permittivity with current injection has been taken into account, no temperature effect was added. The position of the minimum of the response curve as the centroid calculation used for experimental measurements also do not exhibit any variation in their respective value.

Thus, the current injection does not impact the plasmonic response and only the temperature effects will do, similar to all other surface plasmon resonance measurements under variable temperature conditions.

#### 3.2.2. Temperature

The temperature increase that is linked to the current injection, Δ*T*, is inserted using a second-order polynomial for the tendency curve of data represented in Figure 8 (data in Figure 8 represents the maximum value of the temperature profile that is shown in Figure 7):

$$∆T=H_{3}\times I^{3}+\;H_{2}\times I^{2}+H_{1}\times I\;$$

where *H*_3_ = 2.0425 × 10^−7^, *H*_2_ = 4.8311 × 10^−4^, *H*_1_ = −2.4530 × 10^−2^, Δ*T* is in °C and I in mA for a water sensed medium (for an air medium, the parameters are slightly different: *H*_3_ = 2.4045 × 10^−7^, *H*_2_ = 4.6871 × 10^−4^, *H*_1_ = −2.0991 × 10^−2^). 

The temperature effect has been incorporated by its impacts on film thickness and material permittivity.

The thermal expansion of the metallic film has been inserted by using a modified thermal expansion coefficient, α’, reflecting the fact that the film only expands in the direction perpendicular to the sensor surface [40]:

$$\alpha^{\prime }=\alpha \times \frac{1+\upsilon }{1-\upsilon }$$

where υ is the Poisson’s ratio of the material. For thin-film gold layers, this ratio has been measured to be 0.45 [41], which is slightly higher than the usual one for bulk material (0.42). The value of α for gold is classically taken as 14.2 × 10^−6^/°C.

The temperature dependence of the gold refractive index has been introduced from data reported in [16]:

$$\frac{d\left(Re\left(n\right)\right)}{dT}=3.408\times 10^{-1\;}K^{-1}\;\mathrm{and}\;\frac{d\left(Im\left(n\right)\right)}{dT}=-1.381\times 10^{-1\;}K^{-1}$$

for its real and imaginary part, respectively.

The temperature dependence of H-ZF1 glass refractive index has been taken into account following the dispersion formula given in [42]:

$$∆n\left(\lambda ,\;T_{0}+∆T\right)=\frac{n{\left(\lambda ,T_{0}\right)}^{2}-1}{2\times n\left(\lambda ,T_{0}\right)}\times \left(D_{0}\times ∆T+D_{1}\times ∆T^{2}+D_{2}\times ∆T^{3}+\frac{E_{0}\times ∆T+E_{1}\times ∆T^{2}}{\lambda^{2}-\lambda_{TK}{}^{2}}\right)$$

using the parameters given in [43]:

*D*_0_ = −4.73 × 10^−6^; *D*_1_ = 1.27 × 10^−8^; *D*_2_ = −8.17 × 10^−11^; *E*_0_ = 8.61 × 10^−7^; *E*_1_ = 9.43 × 10^−10^; *λ_TK_* = 0.263

where *T*_0_ = 20 °C, Δ*T* is the temperature difference (versus *T*_0_), and *λ* is the wavelength (in µm).

The dispersion formulation of the refractive index of H-ZF1 glass at *T*_0_ (20 °C) is given by [43]:

$$n\left(\lambda ,\;T_{0}\right)=\sqrt{P_{1}+P_{2}\times \lambda^{2}+P_{-2}\times \lambda^{-2}+P_{-4}\times \lambda^{-4}+P_{-6}\times \lambda^{-6}+P_{-8}\times \lambda^{-8}}$$

using the parameters given in [43]:

P_1_ = 2.636187; P_2_ = −9.7034146 × 10^−3^; P_−2_ = 2.55502623 × 10^−2^; P_−4_ = 1.00610407 × 10^−3^; P_−6_ = −4.2521904 × 10^−5^; P_−8_ = 9.06375385 × 10^−6^

and *λ* is expressed in µm.

The temperature dependence of water refractive index is calculated using the formulation given in [44].

**Figure 11:**
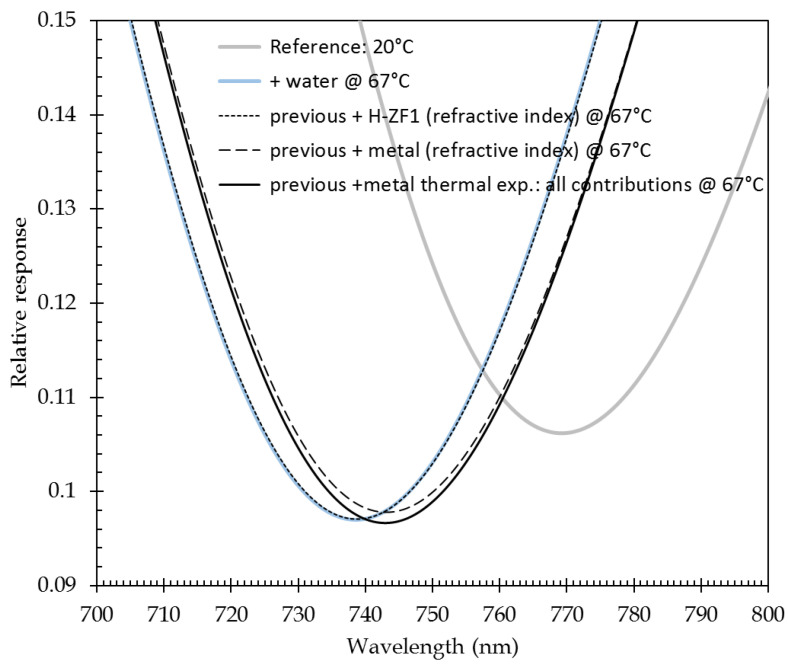
Effect of temperature-dependent parameters on the SPR response curve (here, for a 318-mA injection current, i.e., a 47 °C temperature rise versus ambient). Compared to Figure 10, a zoom on the minimum of the response curve has been made.

Figure 11 summarizes the impact of those different parameters on the plasmonic response. An injection current of 318 mA has been used and a corresponding temperature rise of 47 °C (see Figure 8). The absolute temperature is then 67 °C. Reference response corresponds to that of system at 20 °C including the effect of the injection current on metal permittivity, i.e., the response curve that is presented in Figure 10. In order to determine the impact of each of the temperature-dependent parameters on the response curve, they have been gradually added in the modeling, starting from the reference case:

- temperature of water has been set to 67 °C (curve “previous + water @ 67 °C” in Figure 11);
- value of H-ZF1 refractive index @67 °C is inserted (curve “previous + H-ZF1 (refractive index) @ 67 °C” in Figure 11);
- value of metal refractive index @67 °C is added (curve “previous + metal (refractive index) @ 67 °C” in Figure 11);
- thermal expansion of metal films is inserted (curve “previous + metal thermal expansion: all contributions @ 67 °C” in Figure 11).

The SPR response curve minimum position for all those cases is reported in Table 2.

The insertion of hot water obviously entails a huge blue shift of the response curve. Taking into account the temperature dependence of material refractive index implies red shifts of the response curves. The largest one, more than 4 nm, is linked to the change of refractive index of the metals constituting the plasmonic layer. The effect of glass refractive index variation is much lower, around 0.17 nm. The effect of metallic film thermal expansion implies a blue shift, 0.16 nm. Those latter effects almost compensate for this value of injected current. Contrary to what has been modeled for an angular interrogation scheme [15], no response widening is obtained when increasing the temperature.

In summary, all of the response curve modifications that are observed are due to the temperature. No perceptible influence of current injection, which is the novelty proposed here, has been pointed out. That is to say that, whatever the heating process is, using a usual temperature-controlled enclosure or the localized heating process proposed here, the response curves are identical and the same de-embedding and data processing techniques shall be used. 

## 4. Characterization of Thermal Behavior

### 4.1. Measurement Set-Up

**Figure 12:**
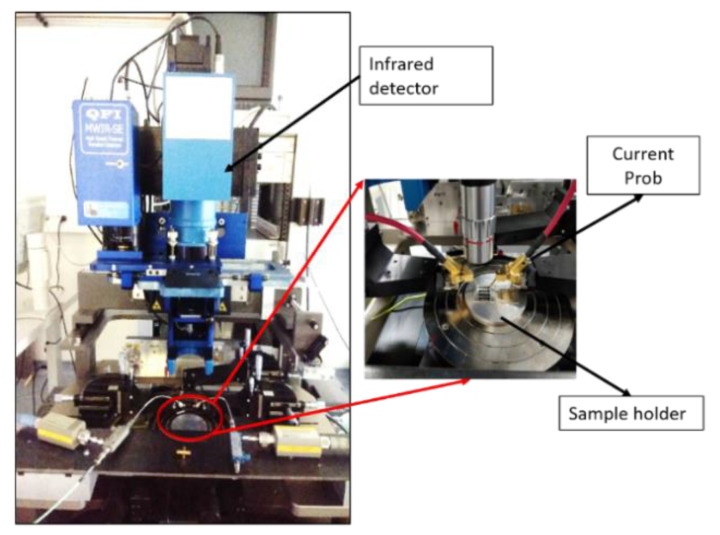
MWIR temperature microscope and measurement set-up.

**Figure 13:**
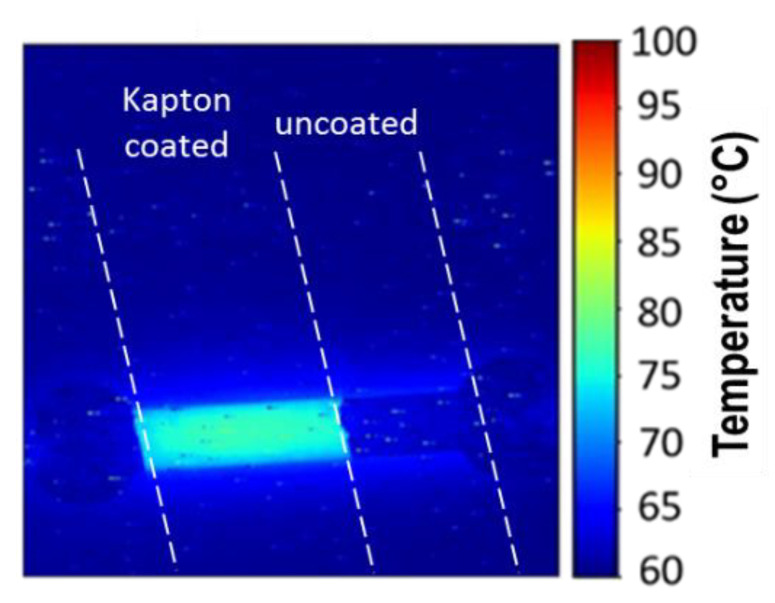
Effect of Kapton coating on thermal IR measurement.

An InfraScope™ MWIR (Medium Wavelength InfraRed) Temperature Mapping Microscope (Quantum Focus Instruments Corporation) was used for measuring the temperature of the channel under current injection. The sensor is placed onto the sample holder of the equipment (Figure 12). To improve the sensitivity of the measurement:

- This holder is maintained at 60 °C. The number of emitted photons for 1° temperature rise is then higher at a base temperature of 60 °C than at 20 °C since it increases as a cubic function with absolute temperature [45].
- A 50-µm thick adhesive polyimide film (Kapton) is placed onto the sensor to enhance emissivity. The evaporated gold thin films, which can be considered as polished gold, have a low emissivity coefficient value; a commonly agreed value is between 0.01 and 0.02, while the value of Kapton is between 0.75 and 0.85 [46]. The difference can be clearly observed in Figure 13, where the left part of the channel has been covered by a Kapton film and the right part is a nude gold surface: no detectable signal is discerned from the uncoated part of the channel.

A 1× magnification lens was used to focus on the sensor channel. The current was injected in the channel using electrical probes placed on both ends (i.e., circular patterns on picture of Figure 2). QFI temperature mapping software was used to perform emissivity correction and to generate the true temperature map. Without injecting a current, the captured image is stored as the reference level.

### 4.2. Thermal Measurements 

**Figure 14:**
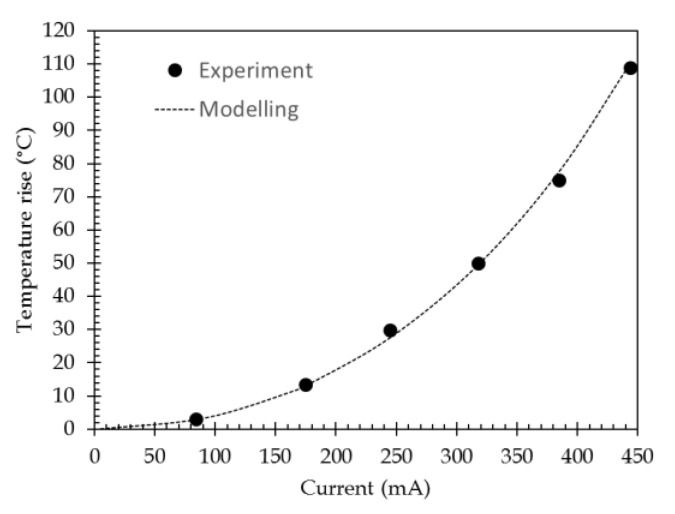
Comparison between the modeled and experimental temperature rise values at the channel center versus the injected current.

The temperature at the channel center has been reported and compared to the modeling corresponding to the case with Kapton covering (Figure 14), since experimental measurement need to use the Kapton coating. A good agreement is obtained on the full range of interest.

**Figure 15:**
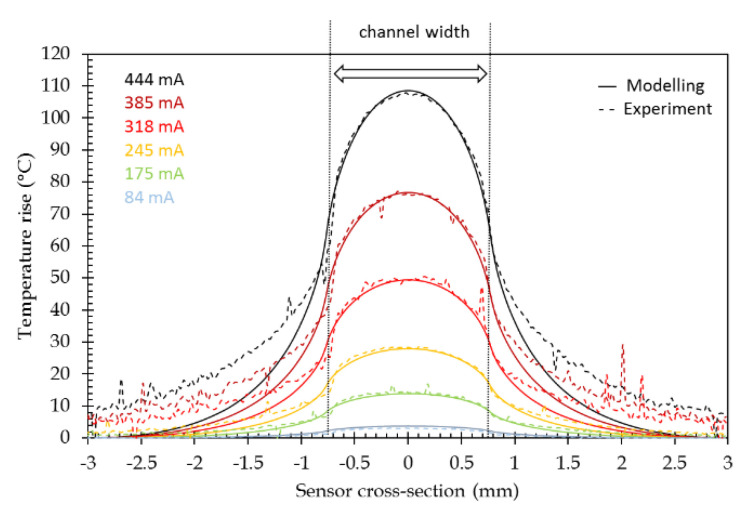
Comparison between the modeled (- - -) and experimental (^___^) cross-sectional temperature rise.

**Figure 16:**
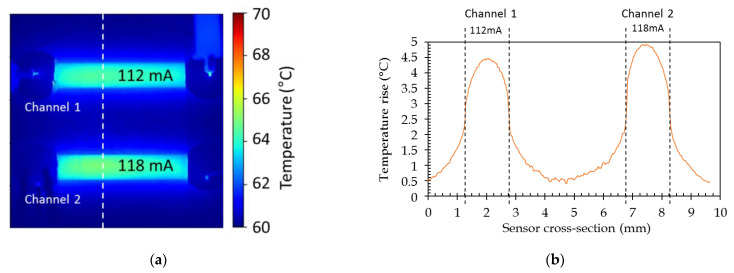
Control of the temperature by injecting two slightly different currents in two adjacent channels: (**a**) thermal camera measurements; (**b**) cross-sectional temperature profile (relative position).

As for the modeling, the temperature profile across the channel has been recorded. The comparison between the modeled and experimental cross-sectional temperature profiles (Figure 15) shows that profiles are in good agreement within the channel width since the experimental ones exhibit a lower temperature decrease outside the channel width. This can be explained by a too low heat capacity (Cp) value for glass in the modeling. Moreover, this divergence grows with the temperature level. This could, then, be related to the non-inclusion of the variation of the heat capacity value of glass with temperature in the simulation. No specific data have been found in the literature for H-ZF1 glass, but the increase of the heat capacity value with the temperature has been reported for BK7 glass [47]. In order to validate the independent temperature control of two adjacent channels, two slightly different currents have been injected in two of them (Figure 16a). It has to be noted that the base temperature of thermal IR imaging is 60 °C (see Section 4.1). On the lateral temperature profile, it can be noticed that a maximal temperature difference of 0.5 °C can be achieved that validates the possibility to separately adjust the temperature of adjacent channels (Figure 16b).

**Figure 17:**
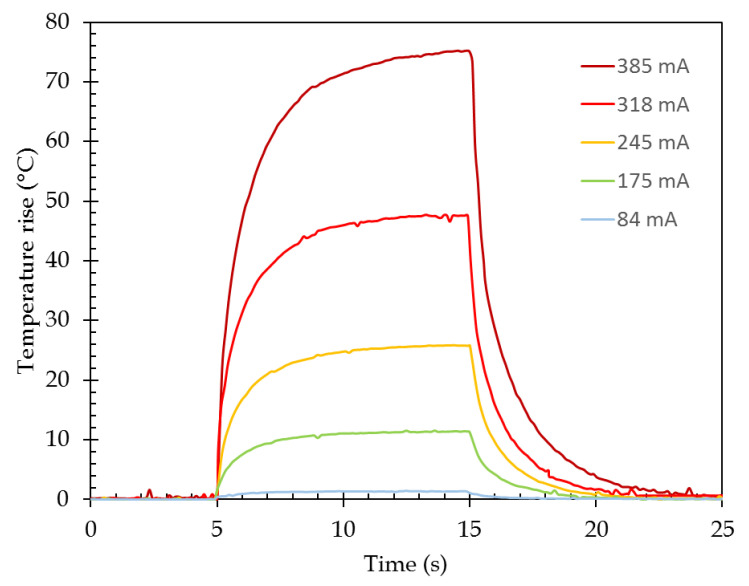
Experimental time response. The current pulse is applied between 5 and 15 s.

A current pulse of 10 s has been injected in order to determine the rise and fall times (Figure 17). Those two have been measured as identical around 3 s ± 10%. The highest values are recorded for higher current values. 

## 5. Thermal Measurements on SPR Experiment

**Figure 18:**
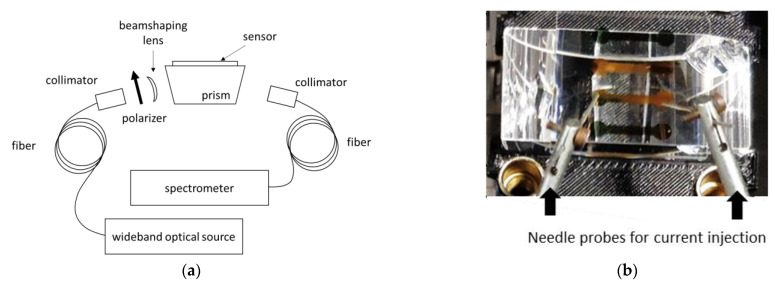
Spectral interrogation scheme based SPR set-up: (**a**) schematic view; (**b**) detail on the current injection using two needle probes.

Our experimental SPR set-up uses a spectral interrogation scheme in a rather usual implementation. It is based on a classical Kretschmann configuration. The prism is made of H-ZF1 glass as the sensor. Sensor and prism are put in close contact using a refractive index fluid from Cargille. The light source is a broadband (360–2600 nm) fiber pigtailed device (SLS201L from Thorlabs). At fiber output, a collimator allows achieving a parallel beam. A polarizer and a beam-shaping lens are used in free space optics to get a TM-polarized line-shaped beam. The line-shaped beam allows to illuminate the four channels. The selection of channel is made by the alignment of the output collimator. The reflected beam is then sent to a compact CCD spectrometer (500–1000 nm, CCS175 from Thorlabs) (Figure 18a). Two needle probes have been used in order to inject the current in the channels. They have been inserted into micropositioners in order to adjust their position over the sensor (Figure 18b).

**Figure 19:**
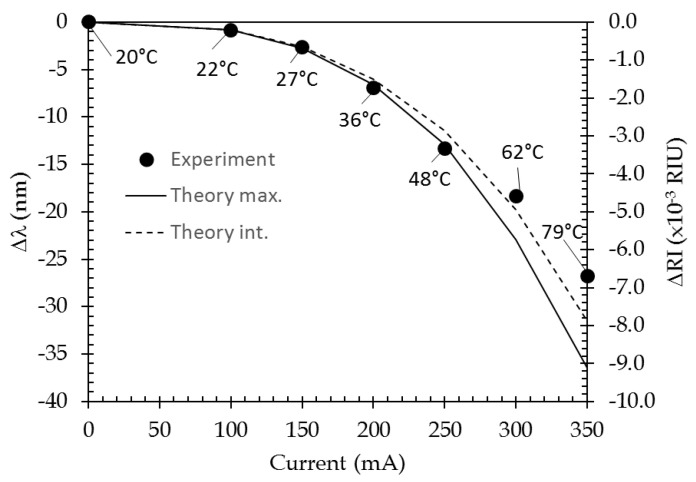
Wavelength shift versus the injected current.

For the experiment, a deionized water drop was deposited over a channel and a current was injected into the plasmonic layer. The response curves were filtered and post-processed using a centroid calculation. The centroid value obtained at 20 °C, λ_0_, acts as a reference value against all subsequent values obtained under current injection, λ*_i_*. The wavelength shift, Δλ = (λ*_i_* − λ_0_) is reported in Figure 19 for each value of the current that has been injected. The different injected currents and corresponding temperatures rises are reported in Table 3. Temperature rise values are the one at the channel center and are issued from Figure 8. The absolute temperature is obtained by adding the room temperature (20 °C) to those values (Table S1, Figure S1).

For the modeling, the water refractive index for each absolute temperature value is calculated from the data given in [44] and the difference versus its value at 20 °C is referenced as ΔRI (see Table 3).

The theoretical curve that is shown in Figure 19 corresponds to the results of the model presented in Section 3.2, i.e., the transfer matrix algorithm where all temperature-dependent contributions are taken into account. 

The same post-data treatment (centroid calculation) has been made on both datasets. Filtering has not been applied to theoretical results since they are obviously exempt from noise. 

The “Theory max.” curve has been calculated for each current at the maximum temperature rise, given in Table 3. A good agreement is observed up to 250 mA, i.e., roughly a 30 °C temperature rise. Nevertheless, some deviation of experimental points to that curve are recorded for high current injection levels. As can be seen in Figure 7 and Figure 15, the higher temperature rises, the less homogeneous the temperature over the channel width is and the more bell-shaped its profile. The channel width has been then discretized into 10 sections in order the bell-shaped temperature profile to be taken into account. A temperature value has been assigned to each section following a temperature profile that corresponds to that current. The overall response has been obtained by the weighted sum of the 10 elementary response curves. The result of this modeling is indicated on "Theory int." curve in Figure 19. 

Even if some discrepancy is still observed for the high current levels, taking into account the bell-shaped temperature profile allows a better fit between theoretical and experimental results.

This remaining discrepancy could be due to experimental parameters that have not been taken into account in the modeling:

- temperature rise can cross over the 1-mm thickness of sensor. In Figure 15 and for high current injection levels, it can be seen that lateral spreading of the temperature rise is not negligible, up to a couple tens of degrees, at 1 mm away from the channel edge. In a first approach, we can consider that this spreading is isotropic within the glass substrate and that such a temperature rise can be obtained on its bottom side. Such a temperature rise can then affect the matching index fluid that is used between sensor and prism. Its temperature increase will modify its refractive index and can, thus, modify the beam injection angle;
- the heat transfer can increase, decreasing the temperature in the channel. This could be linked to the phenomenon that is observed, for an air environment, in Figure 15 for high current injection, where the experimental temperature profile outside the channel does not follow the theoretical one. This hypothesis has not been investigated yet, and it might be especially important for a fluid environment.

Both of those effects cannot be observed during the calibration of the system (Section 4.1) under MWIR temperature imaging, since no optics, and thus, no optical fluid, is used and experiments are done in an air environment. 

**Figure 20:**
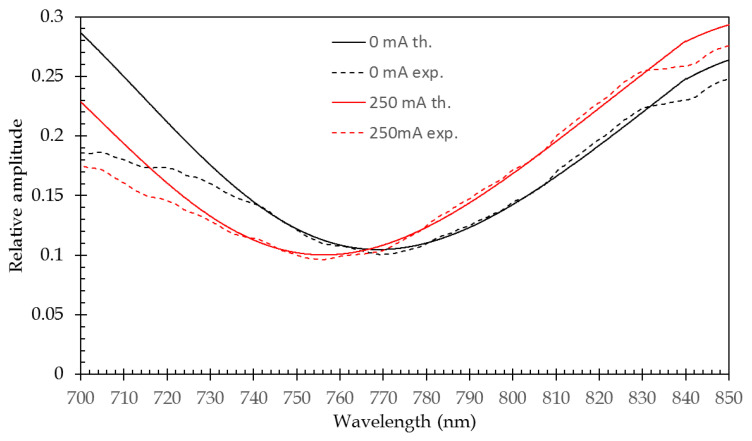
Comparison of experimental and theoretical response curves at 0 and 250 mA current injection levels.

Figure 20 shows the comparison between experimental and theoretical responses for 0 mA and 250 mA injection currents. The minimum position and curve shape are in fine agreement. It has to be noted that the discrepancy that is observed in the lower and higher wavelength ranges are due to the response of the experimental system, which is bell-shaped over the 700–850 nm wavelength range. Here, we chose to represent the 250 mA current injection case, since it is the highest level of injection current that gives matching results between experimental and theoretical responses (see Figure 19).

Some complementary information about the evolution of the characteristics of the SPR measurement, i.e., Full Width at Half maximum (FWHM) of the response curve, sensitivity (S), and Figure of Merit (FOM), with temperature, is given in the supporting information. However, as the injection of current does not produce any modification of the response curve (see Figure 10), those evolutions are identical regardless of the heat that is applied to the system. In other words, using the heating process that is described herein does not involve any supplementary data processing compared to other means of temperature control Figure S2–S4.

## 6. Conclusions

Taking advantage of the very low penetration depth of surface plasmon resonance measurements, we used the metallic layer that is used to create the surface plasmon resonance sensor as a localized heater. The heating technique exploits the Joule effect for controlling the temperature. To achieve such a function, the metallic layer shall be patterned in order to localize the heating areas and the corresponding detection zones. This allows controlling the spatial temperature distribution over the whole sensor surface: a four-channel sensor has been fabricated for experimental validation of the concept. 

The electrical properties of this particular thin film have been measured in order to be used in a COMSOL thermal simulation of such a heating system. The thermal simulation has been conducted implementing both air- and water-sensed media. Air is needed to correlate the simulation with the thermal IR measurements that have been carried out to validate the temperature rise values due to the Joule effect. In order to mimic a biosensing application, water has been used since its optical refractive index is close to the solutions that are used, and its temperature variation is well known. Modeling results obtained using air as a sensed medium are in good agreement with the experimental results obtained under MWIR thermal imaging.

The modeling of the surface plasmon resonance phenomenon has been conducted using a rather usual transfer matrix algorithm. The temperature-current law that was previously determined by the thermal analysis of the Joule heating process has been used to introduce the variations of materials properties with current, and so temperature: the refractive index and thermal expansion of the metals constitute the plasmonic layer and the refractive index of the glass substrate and of the sensed medium. The effect of the injected current has been introduced into the calculation of metal permittivity via a Doppler frequency shift applied in the Drude model. No perceptible effect of the injected current on the plasmonic response curve has been observed for the used current densities. The influence of all above-mentioned parameters has been pointed out, with the main one being linked to the change of the refractive index of metals with temperature. That is to say that the proposed Joule heating process does not involve complementary de-embedding process compared to that used for the other techniques of a temperature-dependent SPR analysis. 

We also demonstrate that as far as an adequate channel spacing versus the temperature range that is used is provided, the temperature of two adjacent channels can be controlled separately without any interference; as an example, a temperature difference of 0.5 °C between two adjacent channels has been reported.

Considering water, a very good agreement has been obtained for temperature rises up to some tens of degrees, which is sufficient for conducting molecular interactions under controlled temperature. Nevertheless, for higher temperature rises, some discrepancy is observed, the source of which shall require further investigation.

In order to fully control, and thus, regulate the temperature, the integration of an embedded temperature sensor on the plasmonic surface is currently being undertaken in order to monitor the real-time temperature of the sensed medium, and thus, to provide the possibility of integrating a temperature control loop. Moreover, such a control will be necessary in case of microfluidic integration, since the fluidic flow and even the flow rate will completely modify the steady-state temperature behavior that has been characterized herein.

Using the plasmonic layer as a heater can then provide:

- an inherently localized temperature control;
- a versatile approach: the design of the channels can be matched to any implementation constraint. Calibration of the heating system will, nevertheless, be dependent on the design;
- a quick temperature control: a response time of a few seconds has been measured;
- a low-cost solution controlling the temperature, since no particular external system is needed.

It can be used during SPR measurements either to follow interactions under temperature gradients or to check their behavior under or following heat pulses. 

## Figures and Tables

**Table 1:** Material parameters used in the COMSOL simulation.

	Glass	Gold	Unit
Density (ρ)	2730	17,000	kg/m^3^
Thermal conductivity (κ)	0.85	300	W/(m.K)
Heat capacity (Cp)	730	128.8	J/(Kg.K)
Electrical conductivity (σ)		From measurement (see Section 2.2)	S/m

**Table 2:** Position of the response curve minimum following the gradual insertion of temperature-dependent parameters (rise of 47 °C) in the modeling.

Parameters Inserted in the Modeling	Minimum Position (nm)	Variation of Minimum Position Versus Reference Case (nm)	Relative Variation (nm)
Reference (all the system is at 20 °C) but current is injected	769.23	-	-
Added: refractive index of water @ 67 °C	738.57	−30.66	-
Added: refractive index of H-ZF1@ 67 °C	738.74	−30.49	+0.17
Added: refractive index of metals @ 67 °C	743.11	−26.12	+4.37
Added: metal thermal expansion @ 67 °C (All contributions)	742.95	−26.28	−0.16

**Table 3:** Driving currents, corresponding temperature rises, and water refractive indices.

Current (mA)	Temperature Rise (°C)	Absolute Temperature (°C)	Water Refractive index (RIU) [43]	ΔRI (10^−3^ RIU) (Versus Reference at 20 °C)
0	0	20	1.326440	0.000
100	1.74	21.74	1.326290	−0.149
150	7.00	27.00	1.325783	−0.657
200	15.95	35.95	1.324732	−1.707
250	27.64	47.64	1.323039	−3.401
300	41.84	61.84	1.320557	−5.883
350	59.05	79.05	1.317045	−9.395

## Supplementary Materials

The following are available online at https://www.mdpi.com/1424-8220/21/6/2035/s1, Figure S1: Calibration of the SPR measurement set-up using reference solutions (@ 20 °C) made of different concentrations of ethylene glycol solutions (% EG by weight in water indicated above each data point). Figure S2: Evolution of the FWHM of the plasmonic response with temperature (modelling), Figure S3. Evolution of the sensitivity with temperature (modelling), Figure S4. Evolution of the Figure of Merit with temperature (modelling), Table S1: Calculated refractive index of EG solutions versus their concentration (%weight) in DI water.
