# Supplementary material for: Plasmonic Layer as a Localized Temperature Control Element for Surface Plasmonic Resonance-Based Sensors
Source: Sensors (Basel). 2021 Mar 13;21(6):2035. doi: 10.3390/s21062035 (PMC8001950; doi:10.3390/s21062035)
Supplement: Supplementary file 1 [file sensors-21-02035-s001.pdf]

sensors

MDPI

Supporting information

Calibration of system sensitivity

Before carrying the thermal measurements, a calibration of the set-up is made using various concentrations of ethylene glycol (EG) solutions (% by weight) in deionized (DI) water at room temperature (20°C). The refractive index of such solutions is well-known and it is given in Table S1 [49].

Table S1. Calculated refractive index of EG solutions versus their concentration (%weight) in DI water (from [46]).

% weight EG in water	Refractive index	Refractive index difference versus reference (x 10^{-6})
0 (reference)	1.33297	0
0.1	1.33306	90
0.4	1.33334	370
0.6	1.33353	560
0.8	1.33371	740
1	1.33390	930

A drop of each solution is set on the SPR bench and the raw results of the plasmonic responses are filtered before a centroid calculation is made to determine the position of the plasmonic peak (same centroid algorithm is used for theoretical results). Using water solution (0%EG solution), the reference position of the plasmonic peak is set. All subsequent results are then turned into relative shifts, Δλ, versus this reference position (Figure SF1). The experimental sensitivity value is then determined as of 3800±100 nm/RIU (RIU: refractive index unit; 1 RIU = refractive index difference of 1) at room temperature of 20°C.This value has been positively compared to the theoretical one that is 3800nm/RIU at 20°C. Sensitivity value has been calculated for higher temperatures and its value decreases with temperature, e.g. 3760, 3530 and 3230 nm/RIU for respectively 25°C, 50°C and 75°C (see Figure S1).

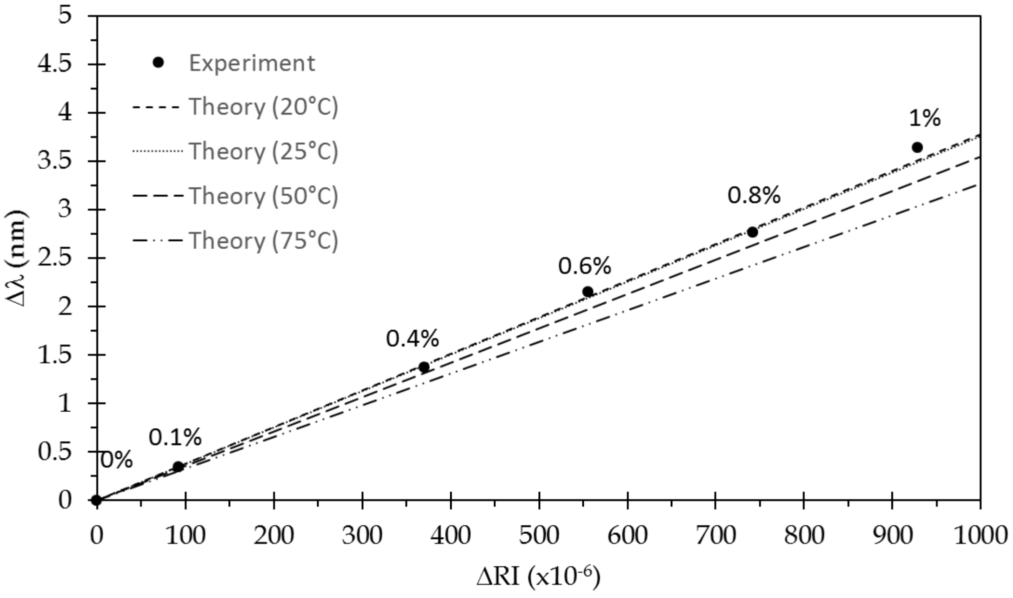

Figure S1. Calibration of the SPR measurement set-up using reference solutions (@ 20°C) made of different concentrations of ethylene glycol solutions (% EG by weight in water indicated above each data point). ΔRI is the refractive index difference of the solutions to pure deionized water (0%) and Δλ is the shift of plasmonic response obtained by centroid calculation.

Evolution of SPR response curve characteristics with temperature

Modelling has been used to quantify the evolution of the characteristics of the SPR measurement, i.e. Full Width at Half maximum (FWHM) of the response curve, sensitivity (S) and Figure of Merit (FOM), with temperature. The latter is the ratio of the two first ones, i.e S/FWHM. Figures S2, S3 and S4 summarize those data.

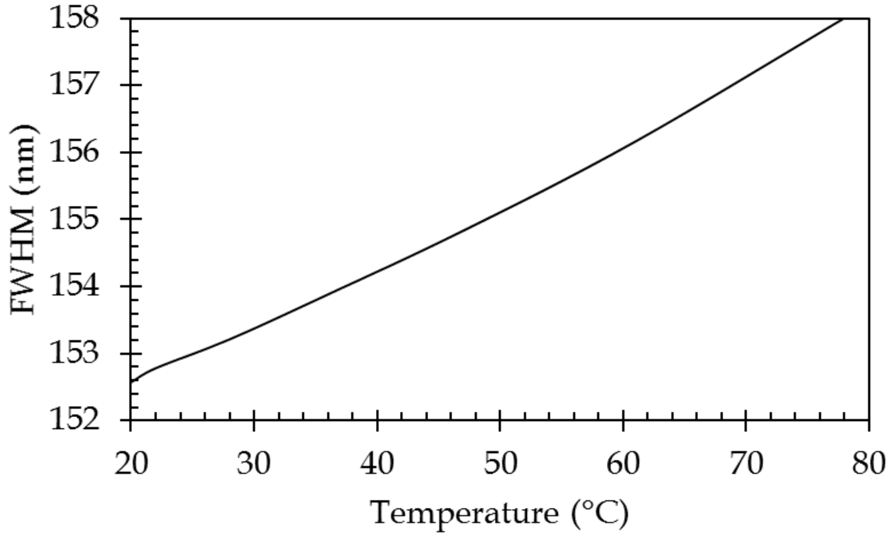

Figure S2. Evolution of the FWHM of the plasmonic response with temperature (modelling)

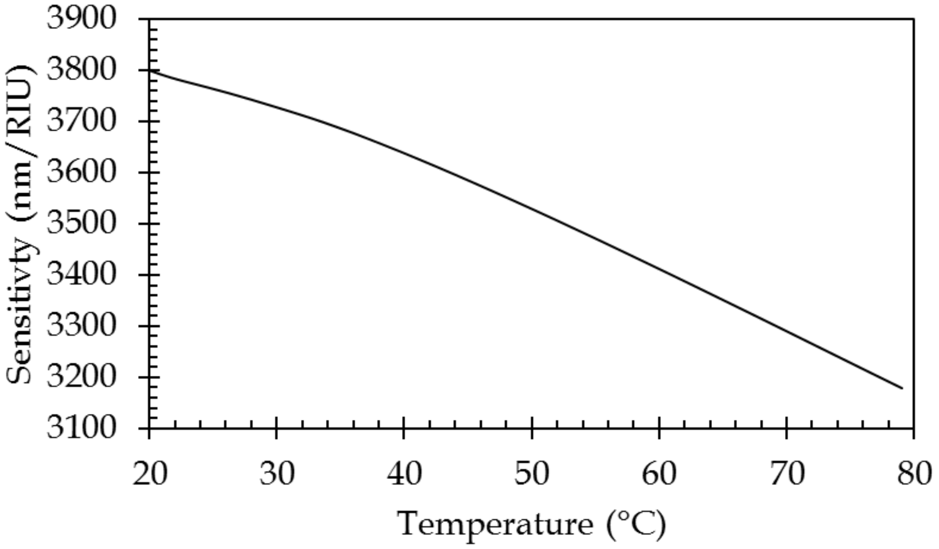

Figure S3. Evolution of the sensitivity with temperature (modelling)

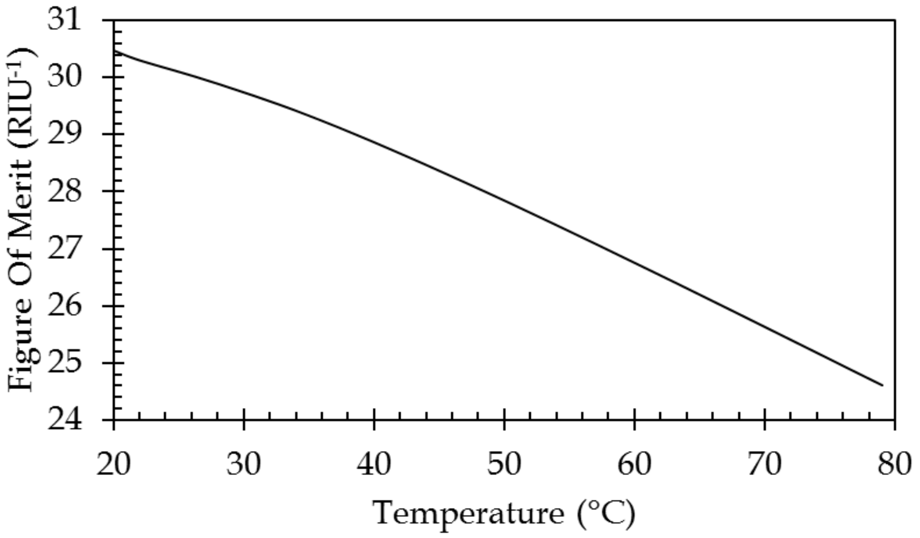

Figure S4. Evolution of the Figure of Merit with temperature (modelling)

The FWHM slowly increases, around 0.1nm/°C, with the temperature. Sensitivity and FOM decrease by, respectively 10nm/RIU/°C and 0.1 /RIU/°C. The major impact on the FOM of the measurement is then linked to the sensitivity decrease with temperature. It has nevertheless to be noted that those behaviors depend only on temperature, whatever it is ambient or provided to the system by any means.
